# Video consultation during follow up care: effect on quality of care and patient- and provider attitude in patients with colorectal cancer

**DOI:** 10.1007/s00464-020-07499-3

**Published:** 2020-03-20

**Authors:** Esther Z. Barsom, Marilou Jansen, Pieter J. Tanis, Anthony W. H. van de Ven, Marjolein Blussé van Oud-Alblas, Christianne J. Buskens, Willem A. Bemelman, Marlies P. Schijven

**Affiliations:** 1 Department of Surgery, Amsterdam UMC, University of Amsterdam, Amsterdam, The Netherlands; 2 Department of Surgery, Amsterdam Gastroenterology and Metabolism, Amsterdam UMC, University of Amsterdam, Amsterdam, The Netherlands; 3 Department of Surgery, FlevoHospital Almere, Almere, The Netherlands; 4 Department of Surgery, Amsterdam Gastroenterology and Metabolism, Amsterdam UMC, Location AMC, Room: G4-133.1, Meibergdreef 9, 1105 AZ Amsterdam, The Netherlands

**Keywords:** Video consultation, Satisfaction, Virtual visit, Surgery, Telemedicine, Colorectal cancer, eHealth, Patient preference, Shared decision making

## Abstract

**Background:**

Video consultation (VC) is gaining attention as a possible alternative to out-patient clinic visits. However, little is known in terms of attitude, satisfaction and quality of care using VC over a face-to-face (F2F) consultation. The aim of this observational survey study was to compare the attitude and satisfaction with VC amongst patients suffering from colorectal cancer and their treating surgeons at the outpatient surgical care clinic in a tertiary referral centre.

**Methods:**

A patient-preference model was chosen following the concept of shared decision making. A total of fifty patients with colorectal cancer were asked to choose between VC- or a F2F-contact during their follow up at the outpatient surgical care clinic and were subsequently assigned to either the VC-group or the F2F-group. Attitude and satisfaction rates of both groups and their surgeons were measured using a questionnaire administered immediately after the consultation.

**Results:**

Out of the 50 patients, 42% chose VC as their preferred follow-up modality. Patients demographics did not differ significantly. Patients who use video calling in their personal life choose VC significantly more often than patients lacking such experience (*p* = 0.010). These patients scored high on both the attitude- and satisfaction scale of the post-VC questionnaire. Patients who chose a F2F-contact seemed to question the ability of the surgeon to properly assess their healthcare condition by using a video connection more (*p* = 0.024). Surgeons were highly satisfied with the use of VC.

**Conclusions:**

Based on patient preference, VC is equivalent to a F2F consultation in terms of patient satisfaction and perceived quality of care. Shared decision making is preferred with regard to which contact modality is used during follow up. For easy uptake in other environments it is to be recommended to facilitate VC using the electronic patient portal.

**Electronic supplementary material:**

The online version of this article (10.1007/s00464-020-07499-3) contains supplementary material, which is available to authorized users.

In the Netherlands, medical care requiring specialist expertise is more and more concentrated in centres of excellence [1]. As a consequence, the travelling distance to the hospital for patients and relatives is increased [2]. Travelling to the surgical oncology outpatient clinic in the post-operative care trajectory, particularly if not (yet) fully recovered, is physically demanding, costly and time consuming. It may also be rather impractical for many patients having a smooth and trouble-free recovery. To overcome unnecessary travelling while preserving the benefits from face-to-face specialist interaction, video consultation (VC) might be an option for many surgical patient in the post-operative surgical care trajectory [3]. The use of video for interaction allows patients to speak and see their caregiver whilst being in the comfort of their own home or workplace [4, 5]. Reported benefits are improved access to healthcare for patients with impaired mobility and enhanced access to care for other informal caregivers, while reducing traveling time and traveling costs preserving patient satisfaction [6, 7].

The use of VC in ambulatory patients undergoing treatment for, or recovering from, colorectal cancer has not been investigated to date according to literature. In patients with colorectal cancer there are no studies reporting on the attitude, use of and/or satisfaction with VC as an alternative for face-to-face (F2F) consultation [8]. Specific concerns may arise on the use of VC for patients with malignant disease, when complex information needs to be shared and when physical aftercare is multidisciplinary and compound [9].

The aim of this study was to determine patient’s preference in choosing the preferred contact modality for regular outpatient follow-up; and to investigate both patient and provider attitude and satisfaction towards VC in comparison with traditional F2F-consultation. We aimed to provide insights on attitude and quality and safety of care while delivering care remotely. Therefore the research questions were: (1) What is the patients’ attitude towards the use of video as consultation modality? (2) How do patients in the VC-group rate the usability of the applied VC technology? (3) How do patients having a F2F-consult assess the quality of the surgeon compared to patients having a VC? and (4) How satisfied are surgeons with F2F-consultation compared to VC?

## Material and methods

### Study design

This study is an observational survey study associated with the implementation of VC at a tertiary care academic centre. The study was carried out between June 2017 and August 2017. Patients were allocated to either the VC-group or the F2F-group based on their personal preference. Approval from the institutional ethics review board was obtained prior to the start of the study. A total of 50 patients were included and written informed consent was obtained from all participants.

### Participants

#### In- and exclusion criteria

Patients with a scheduled appointment at the surgical oncology outpatient clinic were considered eligible for study participation. Inclusion criteria were: age 18 years or older and fluent in speaking and writing in Dutch. Exclusion criteria were: patients who had multiple appointments scheduled at the same day in the hospital (for example in multiple disciplines), patients who did not have access to the Internet, patients without a smartphone, tablet or computer with a camera; and patients who did not activate their electronic patient portal.

#### Eligible appointments

Follow-up appointments in which monitoring of overall condition after surgery was to be expected; as well as appointments scheduled for new complaints, for conveying good results and for discussing treatment options in the post-operative care trajectory were considered eligible for the study. Appointments scheduled for an introductory appointment, conveying bad news and appointments in which a physical examination was likely to take place were not considered to be eligible.

### Study protocol

The study-coordinator approached eligible patients by telephone beforehand to inform them about the study. If patients were willing to participate, verbal informed consent was obtained and participants were allocated to either the VC-group or the F2F-group based on their personal preference. Patients in the F2F-group hence received care as usual.

#### VC-group

**Fig. 1:**
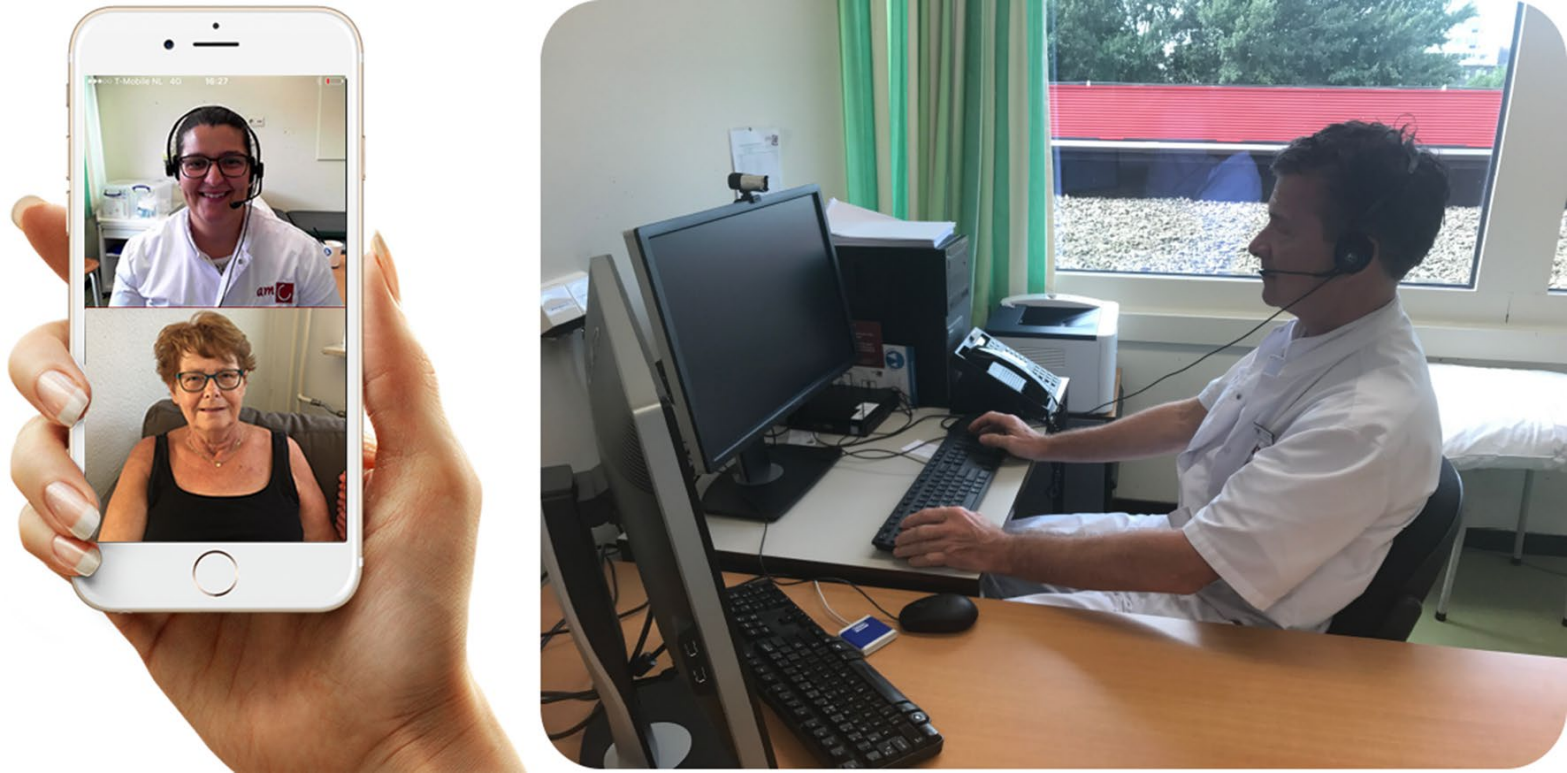
Audio visual connection on a smartphone (patient) and at the workplace (surgeon). Permission of all pictured individuals was obtained

In all patients allocated to the VC-group, a test consult with the study-coordinator was scheduled. During this test consult additional information was provided about the study and the setup and quality of the video connection. When no connection could be established, participants were called by telephone in order to try and coach them through. During the test consult, the System Usability Scale (SUS) was verbally completed. After this, the actual VC with the participant’s caregiver was planned (Fig. 1). Afterwards, participants automatically received a questionnaire via the same electronic patient portal. If there was a need for immediate physical consultation, the patient was instructed to present him or herself to the hospital on an acute base. Information concerning the technical design of our VC system is provided in Supplementary file 1.

#### F2F-group

Patients who preferred a traditional F2F-appointment were seen in person at the outpatient clinic. The study questionnaire was sent digitally after their consult using SurveyMonkey™. This online survey tool was considered to be compliant with privacy legislation as the study was completed before the new General Data Protection Regulation (GDPR) legislation became effective.

### Study measures

#### Patient characteristics

Demographics such as age, gender and diagnosis were retrieved from the electronic hospital record (EHR).

#### Patient attitude towards the use of video as consultation modality

In the absence of a validated questionnaire matching our research population, a study-specific questionnaire (PAT-VC) was designed, adapted from the questionnaire used by Mekhijan et al. [10]. This questionnaire was considered most promising based on our systematic review evaluating the measurement properties of validated questionnaires concerning patient satisfaction with video consultation [11]. The PAT-VC questionnaire consisted of items concerning perceived usefulness, benefits, confidentiality, efficiency and satisfaction. These items were assessed by evaluating various statements on a 5-point Likert-scale (range: ‘totally disagree’ to ‘totally agree’) and by their answers to multiple-choice questions on the frequency of use and experience with online communication. The questionnaire included open questions where patients could elaborate on their given answers.

#### Usability of the used VC technology

To assess the usability of VC, the validated System Usability Scale (SUS) was used [12]. The SUS is an effective tool to measure usability, easy for study participants to comprehend, and provides a single score which is easy to understand and interpret. Based on a 10-item questionnaire, answered on a 5-point Likert scale, it provides a score from 0 (negative) to 100 (positive).

#### Patient satisfaction on interaction with the health care provider

Patients’ satisfaction with the individual performance and professional competence of a provider was measured using the ten-item Multi Source Feedback (MSF) questionnaire for patients, with a 5-point Likert-scale response mode ranging from 1 (‘Totally agree’) to 5 (‘Totally disagree’). The Dutch Federation of Medical Specialists adapted and validated the MSF in Dutch in order to evaluate the quality of the individual performance of medical specialists [13]. The English version shows high reliability, validity, and feasibility in the clinical setting (*α* > 0.90) [14].

#### Provider satisfaction with the consult

The participating surgeons completed a 10-item questionnaire at the conclusion of each consult in both groups. This, to assess satisfaction, benefits and efficiency of the consult. Five items were assessed on a 5-point Likert-scale (range: ‘totally disagree’ to ‘totally agree’), 4 items were yes/no questions and 1 question was open ended.

### Statistics

Data are presented by their means and standard deviations when normally distributed, or by their median and interquartile range (IQR) in case of a non-normal distribution. Categorical data are presented in frequencies and proportions. To test whether there was a difference in baseline characteristics between both groups, the Mann–Whitney U test was performed as data was not distributed normally. Statistical significance was considered when the calculated probability (*p*) was equal to or smaller than 5% (*p* ≤ 0.05). IBM SPSS version 25 was used for statistical analyses (IBM corp., Almond, NY, USA).

## Results

### Participants

During the study period, a total of 103 patients scheduled to visit the outpatient clinic for colorectal cancer were screened for eligibility. In total, 75 (73%) patients were considered eligible. Of these, 6 patients did not meet the inclusion criteria, 14 patients were scheduled for multiple appointments and 5 patients refused to participate.

**Fig. 2:**
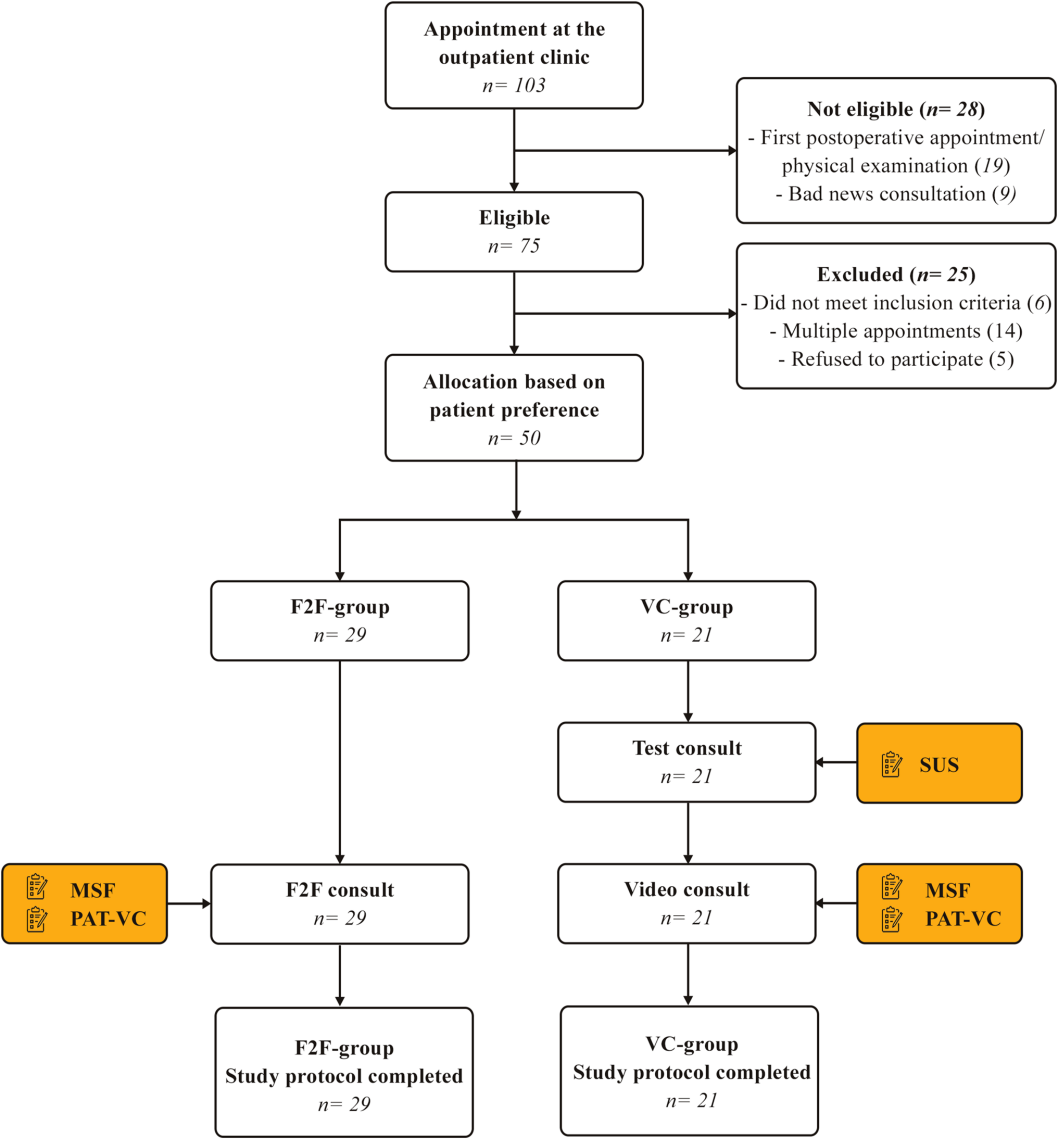
Flowchart of the participants through the study protocol

Out of the 50 patients who were willing and able to participate, 21 patients indicated to prefer a VC. None of the patients were lost to follow up and every patient completed the questionnaires. An overview of the protocol flowchart is described in Fig. 2.

### Patient characteristics

Baseline patient characteristics were similar between groups (Table 1). None of the patients reported to have any previous experience using video as means for interaction (a VC) in healthcare. In both groups, regular follow-up was most often the reason for scheduling a consultation. The reason for follow up in the VC-group was more often to discuss overall progress, whereas in the F2F-group it was to discuss a complaint (*p* = 0.011). Patients who use video calling in their personal life chose a VC significantly more often than patients lacking such experience (*p* = 0.010). Although there was no significant difference, patients choosing VC tend to live further away from the hospital. Details of patient characteristics are summarized in Table 2.

**Table 1:** Demographic characteristics of study sample

Patient demographics	F2F-group (*n* = 29)	VC-group (*n* = 21)	*p*-value
Age in years, mean (SD)	68 (57–74)	61 (53–69)	0.089^#^
Gender, *n* (%)			
Male	11 (39.3)	11 (52.4)	0.310^¥^
Ethnicity, *n* (%)			
Caucasian	26 (89.7)	18 (85.7)	0.914^¥^
Southern America	2 (6.9)	2 (9.5)	
Northern Africa	1 (3.4)	1 (4.8)	
Clinical diagnosis, *n *(%)			
Malignancy	23 (79.3)	17 (80.9)	0.793^¥^
Inflammatory disease	6 (20.7)	4 (19.1)	

^#^ Mann Whitney U test, ^¥^Chi Square test

**Table 2:** Patients’ characteristics regarding the (personal) use of video consultation (VC) and travelling expenditures

Patient characteristics	F2F-group (*n* = 29)	VC-group (*n* = 21)	*p*-value
Reason for follow up			
Regular follow-up	18 (62.1)	13 (61.9)	0.011^¥^*
Discussing a complaint	4 (13.8)		
Discussing overall progress		6 (28.6)	
Discussing a result	2 (6.9)		
Discussing a treatment	5 (17.2)	2 (9.5)	
Approximate distance travelled, km (SD)	18.9 (10.2–36.0)	35.6 (16.4–55.8)	0.066^#^
Estimated one-way travel time by public transportation, min (SD)	57 (32–87)	77 (50–100)	0.771^#^
Travelling cost, € (SD)			
By car	2.76 (1.47–6.48)	4.64 (1.87–7.22)	0.185^#^
By train	7.30 (2.42–10.49)	9.17 (4.26–13.1)	0.140^#^
Type of device, *n* (%)			
iPhone	5 (17.2)	–	0.467^¥^
iPad	5 (17.2)	6 (28.6)	
Android phone	10 (34.5)	8 (38.1)	
Android tablet	2 (6.9)	1 (4.8)	
Apple computer	2 (6.9)	2 (9.5)	
PC/desktop	5 (17.2)	4 (19.1)	
Technical experience with VC, *n* (%)			
None	8 (27.6)	1 (4.8)	0.165^¥^
A little	12 (41.4)	7 (33.3)	
Enough	4 (13.8)	6 (28.6)	
A lot	4 (13.8)	5 (23.8)	
Experienced	1 (3.4)	2 (9.5)	
Previous VC in healthcare, *n* (%)	0 (0)	0 (0)	1.000
Private use VC, *n* (%)			
None	8 (27.6)	1 (4.8)	0.010^¥^*
A little	12 (41.4)	7 (33.3)	
Sufficient	4 (13.8)	6 (28.6)	
A lot	4 (13.8)	5 (23.8)	
Experienced	1 (3.4)	2 (9.5)	

^#^Mann Whitney U test, ^¥^Chi Square test, *p < 0.05

### Patient attitude towards the use of video as consultation modality

**Fig. 3:**
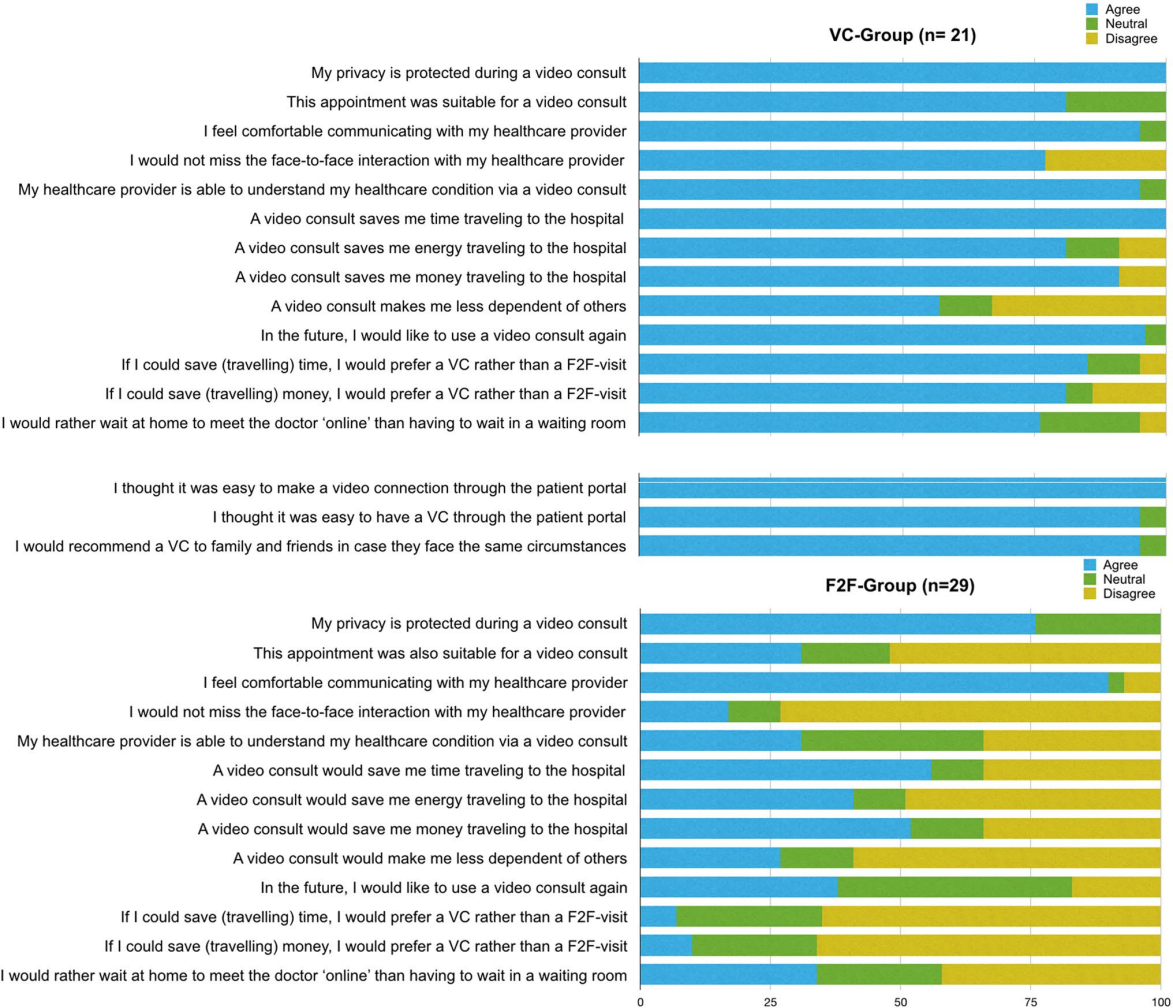
Results of the PAT-VC questionnaire after the VC or F2F-visit. Answers were provided on a 5-point Likert scale and are presented in percentages. Categories ‘Totally agree’ and ‘Agree’ were pooled as ‘Totally disagree’ and ‘Disagree’

Patients’ responses to the patient attitude towards video consulting (PAT-VC) questionnaire are shown in Fig. 3. Overall the patients in the VC-group expressed more confidence in the quality of care over a video connection and expressed a highly positive attitude towards the benefits of VC.

Patients who preferred a VC indicated that they would miss the physical interaction less than patients who preferred a F2F-appointment (33% vs 72%, *p* = 0.001); moreover, they had more confidence in the ability of the healthcare provider to understand their healthcare condition through a video connection (95% vs 31%, *p* = 0.024). Although privacy concerns were not prominent in both groups, the F2F-group reported having less confidence in the safeguarding of their privacy than the VC-group did (76% vs 100%, *p* = 0.020). Almost all patients in the VC-group (96%) indicate they would like to use VC again in the future, as did 11 out of 29 (38%) within the F2F-group.

All patients in the VC-group felt it was easy to make the video connection (100%). None of the patients in the VC-group requested additional F2F follow up after using the VC. In this group, 95% felt it was convenient for the patient to have access to VC via the patient portal and 95% would recommend using VC to family and friends.

Open text field answers describe the following reasons to choose for a VC; saving travelling time and –money and avoiding stressors as busy traffic and waiting in the waiting room.

Patients who were not willing to use a VC gave reasons as the absence of physical contact and the unfamiliarity with using VC in healthcare.

### Usability of the used VC technology

The overall mean score on the System usability scale (SUS) was 85 (SD = 8), which correlates with an excellent grade of usability [15]. Out of all participants, 14/21 (67%) rated the usability as excellent (SUS score > 80.3) and 7/21 (33%) rated the usability as good (SUS score 68–80).

### Patient satisfaction with the quality of the health care provider

**Fig. 4:**
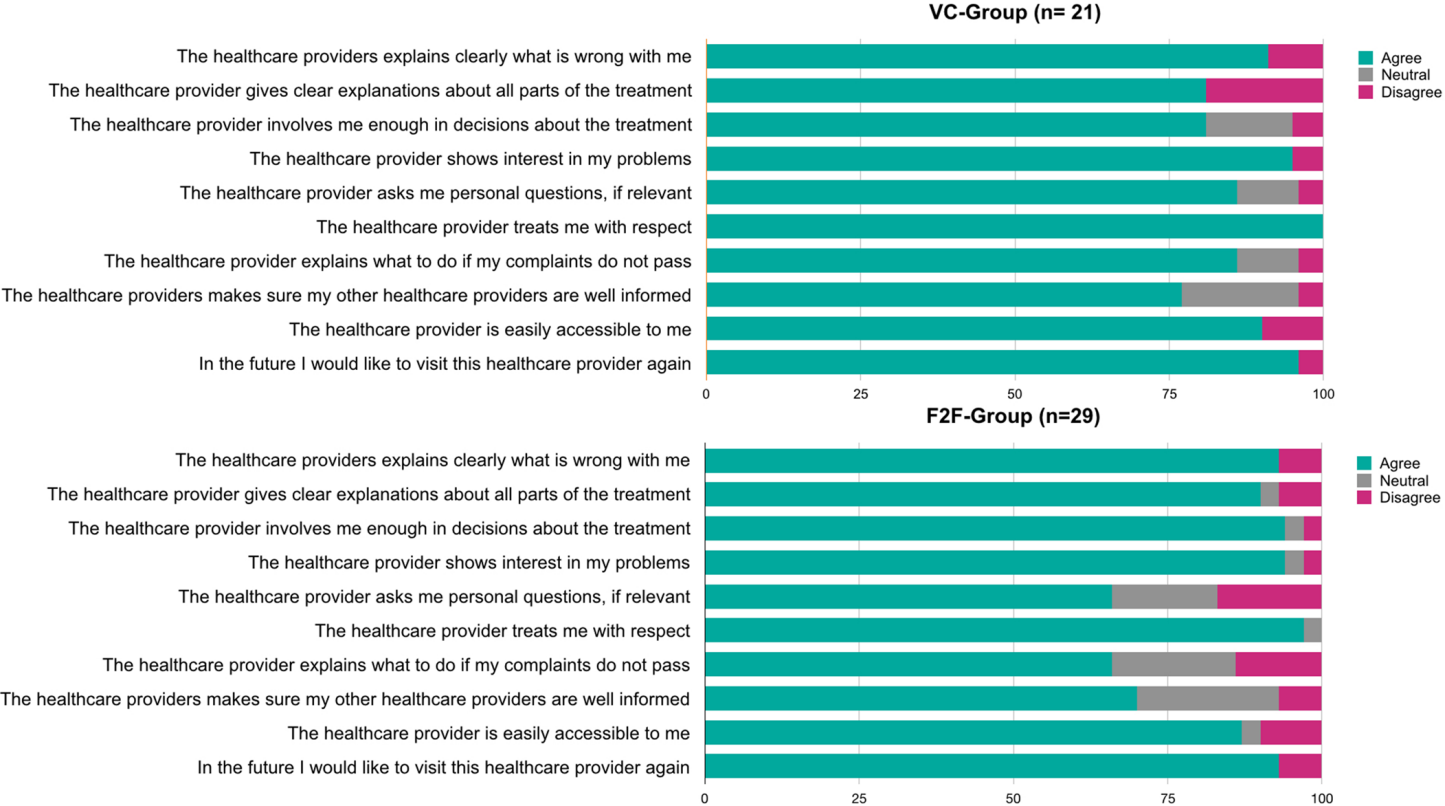
Results of the Multi Source Feedback Questionnaire completed by patients after the VC or F2F-visit. Answers were provided on a 5-point Likert scale and are presented in percentages. Categories ‘Totally agree’ and ‘Agree’ were pooled as ‘Totally disagree’ and ‘Disagree’

The level of satisfaction with the individual performance and professional competence of the healthcare provider as measured by the MSF questionnaire was consistently high in both the VC-group and the F2F-group (Fig. 4). Differences in satisfaction were observed in favour of the VC-consult related to asking personal questions (86% vs 65%, *p* = 0.554) and explaining what to do when complaints do not disappear (86% vs 56%, *p* = 0.440), though not statistically different.

### Provider satisfaction with the consult

The overall grade of satisfaction with VC amongst surgeons was higher (9, IQR 9–10) than for the F2F-consult (8, IQR 7–8). In both groups, surgeons felt they had discussed everything that was necessary in all of the included cases. However, in 13 patients of the F2F-group, the surgeon indicated that a VC would not have been suitable, as in 5 patients it was necessary to perform physical examination during the consultation that was not anticipated considering the nature and timing of the consultation. In 12 out of these 13 patients in the F2F-group, surgeons indicated that they would have missed the physical contact and therefore thought a VC would not have been suitable. However, in none of the patients in the VC-group, surgeons felt that they had actually missed the physical contact after a VC, nor was it necessary to perform physical examination. Moreover, surgeons felt they were able to assess the patient’s condition through a video connection and in none of the patients additional follow up after at the hospital after a VC was necessary. There were no cases of dropped connections or technical difficulties.

## Discussion

This study is, to our knowledge, the first study evaluating the use of VC based on patients’ preferences for follow-up consultation among colorectal cancer patients. Patients preferring VC express a highly positive attitude and satisfaction using VC for their consultation, as did their providers. None of the patients in the VC-group requested further F2F follow up after the VC, confirming VC was sufficient in answering health related issues. This is in accordance with a recent study which shows that the quality of doctor-patient communication did not differ between screen-to-screen contact or F2F-contact [16].

VC needs to be evaluated considering the current debate on how to design the best follow up regiment for colorectal cancer patients. Patient preference needs to have the primary focus in such a debate [17, 18] Giving patients a voice in how they would like to receive care is meaningful to organize patient-centered care, which is an important element of quality of care [19, 20]. Next, in modern healthcare there needs to be a focus on environmental sustainability, including CO_2_ awareness and decrease [21]. Why have patients travelling for specific expertise, when there is often no need for physical interaction and the quality of patient-provider interaction can be maintained using digital solutions such as VC?

A substantial issue considering the latter is also the frequency of currently hospital related visits. Despite the fact that intensive follow up with a high frequency often leads to anxiety and distress amongst patients, patients do belief continued follow up is important [22]. A modality with a lesser burden to both patient and environment, such as telephone consult or VC, must therefore be considered.

Alternative methods to reduce the burden of intensive F2F follow up include telephone follow up, VC and/or remote surveillance [23]. F2F consultation can be reduced by scheduling telephone consultations. However, non-verbal communication is an important aspect of a trusting relationship, which is lacking during a telephone consult [24]. VC does allow for non-verbal communication and offers the additional benefit of receiving care at home.

In literature, computer illiteracy in both patients and healthcare providers is considered to be a possible barrier in using VC [25, 26]. Indeed, within our study population, patients with experience using video calling in daily life seem choose a VC over F2F consultation more often. Patients who were hesitant in choosing a VC express being insecure of using new technology. However, 38% of patients in the F2F-group were willing to give VC with their healthcare provider a try in the future, but state they first want to get familiar using video calling in daily life before using it for health purposes. In contrast to other studies, the usability of our VC system was rated to be excellent [27]. The VC functionality was incorporated into the EHR and electronic patient portal, which is part of the organizational and technological infrastructure in use. Because most patients are familiar with the environment of the electronic patient portal, we believe even patients without technical or VC experience are able to use the VC system. Patient counselling and informative flyers could however increase confidence in patients who need extra support.

The study design of this study is ought to be considered when interpreting its findings. Patients were assigned to either the F2F-group or the VC-group based on the patients’ preference, which introduced selection bias. However, the sample itself was not self-selecting. The researchers could not have predicted which patients and the amount of patients who would choose a VC based on the inclusion criteria. In addition, the percentage of patients who preferred a VC in this cohort is in concordance with the results of a national survey study amongst 987 Dutch patients [28]. Furthermore, VC is implemented as an alternative for F2F consultation upon request. They will not replace all F2F consultations in the future. Therefore, the used study design has a greater external validity if translated to a bigger population.

Future studies should explore which appointments are considered suitable to be replaced by a VC by patients and healthcare providers. Especially with regard to introductory appointments and appointments in which bad news is conveyed and to examine in which cases the lack of physical examination might have implications. In addition, it would be interesting to determine if video consultation could be part of the clinical surveillance, offering a solution to the cost related issues of medical imaging during follow up.

## Conclusion and recommendations

In conclusion, for patients with colorectal cancer, treated at the outpatients clinic of a centre of excellence VC must be considered as a contact modality equivalent to F2F consultation for the majority of appointments. Based on the preference of patients, patients and surgeons approve and are satisfied with this mode of care delivery. Attention should be paid to patients who are willing to use VC but are hesitant because they feel they need support in using VC. If hesitations are addressed and clarified through patient counselling, more patients may benefit from receiving online care in the near future.

Ultimately, the implementation of VC should be imbedded into a hospital’s EHR in order to allow the most sufficient workflow for healthcare providers and protect both provider and patient privacy.

## Electronic supplementary material

Below is the link to the electronic supplementary material.

Supplementary file1 (DOCX 13 kb)
