# Supplementary material for: Video consultation during follow up care: effect on quality of care and patient- and provider attitude in patients with colorectal cancer
Source: Surg Endosc. 2020 Mar 20;35(3):1278–87. doi: 10.1007/s00464-020-07499-3 (PMC7886764; doi:10.1007/s00464-020-07499-3)
Supplement: Supplementary file 1 — Supplementary file1 (DOCX 13 kb) [file 464_2020_7499_MOESM1_ESM.docx]

## **SUPPLEMENTARY FILE 1.**

## Video consultation equipment

At the outpatient clinic of the hospital, a consultation room was equipped for VC. Software enabling secure VC connection (VIDYOTM (VIDYO Inc., Hackensack, NJ, USA)) was installed on a hospital computer, integrated with EPIC Hyperspace^TM^ 2017, the electronic hospital record (EHR) of use. For patients, the video connection was accessible via MyChart^TM^, the electronic patient portal of EPIC.

Participating patients in this study could connect to MyChart^TM^ for videoconferencing using a desktop computer, tablet or smartphone. The initiative to start a VC remained with the surgeon. To access and start a VC, surgeons started the two-way audio and visual streaming by clicking the ‘connect to video’ button, within the appointment scheduled in their own outpatient agenda using the EHR. Therefore, no additional IT time is required to set up the VC connection. VC encounters were documented in a customary manner within the EHR. In this phase it was not possible yet to record the VC as our goal was to first establish a successful and safe implementation of real time VC’s. Because the video software was integrated within the EHR, confidentiality was ensured through standardized regulations of the hospital following the prior and current GDPR guidelines (protected personal two-factor verification login portal).
